# Developing a model of best practice for teams managing crisis in people with dementia: a consensus approach

**DOI:** 10.1186/s12888-020-02899-0

**Published:** 2020-10-13

**Authors:** Jennifer Yates, Miriam Stanyon, David Challis, Donna Maria Coleston-Shields, Tom Dening, Juanita Hoe, Kaanthan Jawahar, Brynmor Lloyd-Evans, Esme Moniz-Cook, Fiona Poland, Amy Streater, Emma Trigg, Martin Orrell

**Affiliations:** 1 Institute of Mental Health, Division of Psychiatry and Applied Psychology, School of Medicine, University of Nottingham, Jubilee Campus, Nottingham, NG7 2TU UK; 2 Division of Nursing, City University London, London, UK; 3 Division of Psychiatry, University College London, London, UK; 4 Faculty of Health Sciences, University of Hull, Hull, UK; 5 University of East Anglia, Norwich, UK; 6 Research and Development, North East London NHS Foundation Trust, Ilford, UK

**Keywords:** Crisis resolution team, Dementia, Best practice, Fidelity, Consensus

## Abstract

**Background:**

Teams delivering crisis resolution services for people with dementia and their carers provide short-term interventions to prevent admission to acute care settings. There is great variation in these services across the UK. This article reports on a consensus process undertaken to devise a Best Practice Model and evaluation Tool for use with teams managing crisis in dementia.

**Methods:**

The Best Practice Model and Tool were developed over a three stage process: (i) Evidence gathering and generation of candidate standards (systematic review and scoping survey, interviews and focus groups); (ii) Prioritisation and selection of standards (consultation groups, a consensus conference and modified Delphi process); (iii) Refining and operationalising standards (consultation group and field-testing).

**Results:**

One hundred sixty-five candidate standards arose from the evidence gathering stage; were refined and reduced to 90 through a consultation group exercise; and then reduced to 50 during the consensus conference and weighted using a modified Delphi process. Standards were then operationalised through a clinical consultation group and field-tested with 11 crisis teams and 5 non-crisis teams. Scores ranged from 48 to 92/100. The median score for the crisis teams was 74.5 (range 67–92), and the median score for non-crisis teams was 60 (range 48–72).

**Conclusions:**

With further psychometric testing, this Best Practice Model and Tool will be ideal for the planning, improvement and national benchmarking of teams managing dementia crises in the future.

## Background

United Kingdom (UK) health and care policy is committed to enabling more people with dementia to live longer in their own homes, and fewer unnecessary inpatient admissions [1]. This is underpinned by a desire to maintain independence for people with dementia to improve quality of life [2], and reduce financial costs associated with admission to acute settings [3, 4]. Specific community-based services exist to support people with dementia and their carers during times of crisis when the ability to remain independent is compromised and inpatient admission to hospital likely [5]. Such situations often arise due to a change or deterioration in physical and/or mental health function of the person with dementia, a breakdown in care provision, or issues related to polypharmacy and inappropriate use of anti-psychotic treatments. Teams delivering crisis resolution services for people with dementia and their carers are referred to here as ‘Teams Managing Crisis in Dementia’ (TMCDs). TMCDs are multidisciplinary teams, usually provided by Mental Health Trusts, based in the community as either independent teams or as part of a Community Mental Health Team or Memory Assessment Service. Their typical model of working involves a rapid assessment to establish needs of the person with dementia and carers, most often on the basis of referral from primary care in response to a crisis situation, and an intensive short-term intervention to manage or reduce risk of admission whilst appropriate long-term support is arranged with other community health and social care services.

TMCDs vary greatly in their titles, eligibility criteria, models of working, and approach to crisis management [5, 6]. Neither policy documents nor commissioning guidance provide exact details on how TMCDs, or crisis resolution services for older people or people with dementia, should be designed or implemented [7]. This contrasts with crisis resolution services for working age adults [8], and other mental health services for older people. For example, Memory Assessment Services have clear specifications and can gain accreditation from the Royal College of Psychiatrists (RCP) through demonstrating adherence to agreed standards of good practice [9]. Various national policy documents such as the Dementia Well Pathway [10] and the Prime Minister’s Challenge on Dementia (2015) [11] emphasise the importance of supporting people with dementia and their carers, but do not detail how services should be commissioned to maintain independence at the point of crisis. A lack of established and validated guidelines for TMCDs results in variation in quality and effectiveness, and a postcode lottery of access to services.

Variation in health services delivery can enable services to respond to local needs and the unique demographics of local populations, to provide patient centred care. However, some variation is unwarranted [12], and creates issues such as lack of understanding by other health and social care professionals and the public regarding the remit and eligibility criteria of the team [13], a mismatch between people with dementia and carers’ expectations of what the team can offer, and lack of equitable access for all people with dementia and their carers. A series of agreed standards that underpin how TMCDs deliver their service, and resources to implement practices outlined in these standards, is required to achieve effective, consistent, and high-quality performance and measurement.

The approach followed in this study is similar to established methodologies such as those used by the RCP Centre for Quality Improvement (CCQI), and the National Implementing Evidence-Bases Practices Project. The CCQI leads quality improvement networks across several UK mental health services, developing standards and auditing fidelity to such standards nationally, resulting in accreditation of services. Whilst standards and accreditation exist for Crisis Resolution and Home Treatment Teams, no such standards or audit processes exist for TMCDs. This study also closely followed the methods used in the CORE study [14] which constructed a fidelity scale for adult crisis resolution teams (CRT) in the UK. The CORE study used the following procedure: (i) concept mapping to identify potential characteristics of CRT services from a review of the literature, a national survey, and interviews and focus groups with relevant stakeholders; (ii) an expert panel discussion group to sort the resulting ‘longlist’ of potential components of a CRT model into a set of fewer than 100 statements; (iii) stakeholder meetings, where statements were sorted into groups based on conceptual fit and order of importance in delivering an effective CRT service; (iv) field-testing of the scale during review days with several teams, where psychometric properties of the scale were established.

This work is part of the Achieving Quality and Effectiveness in Dementia Using Crisis Teams (AQUEDUCT) research programme (RP-PG-0612-20,004). AQUEDUCT aims to improve the quality and effectiveness of care for people with dementia experiencing crisis This article describes the development of the Best Practice Model and Best Practice Tool (Work Package 1) [15]. The Model, Tool, and other resources will be trialled in subsequent Work Packages and discussed in future publications. This study used a consensus process to develop a model of best practice encompassing a set of standards for TMCDs to work towards, and a measure to test fidelity to this model.

## Methods

### Design

The Work Package 1 protocol is published elsewhere [15]. An iterative, multi-methods, consensus approach was chosen as it provides pragmatic information where empirical evidence is limited and in exploring ambiguous or controversial topics [16]. The iterative nature allowed new insights to be incorporated into later stages, creating a dynamic and practical design, using the best available evidence at each point.

### Participants

**Fig. 1:**
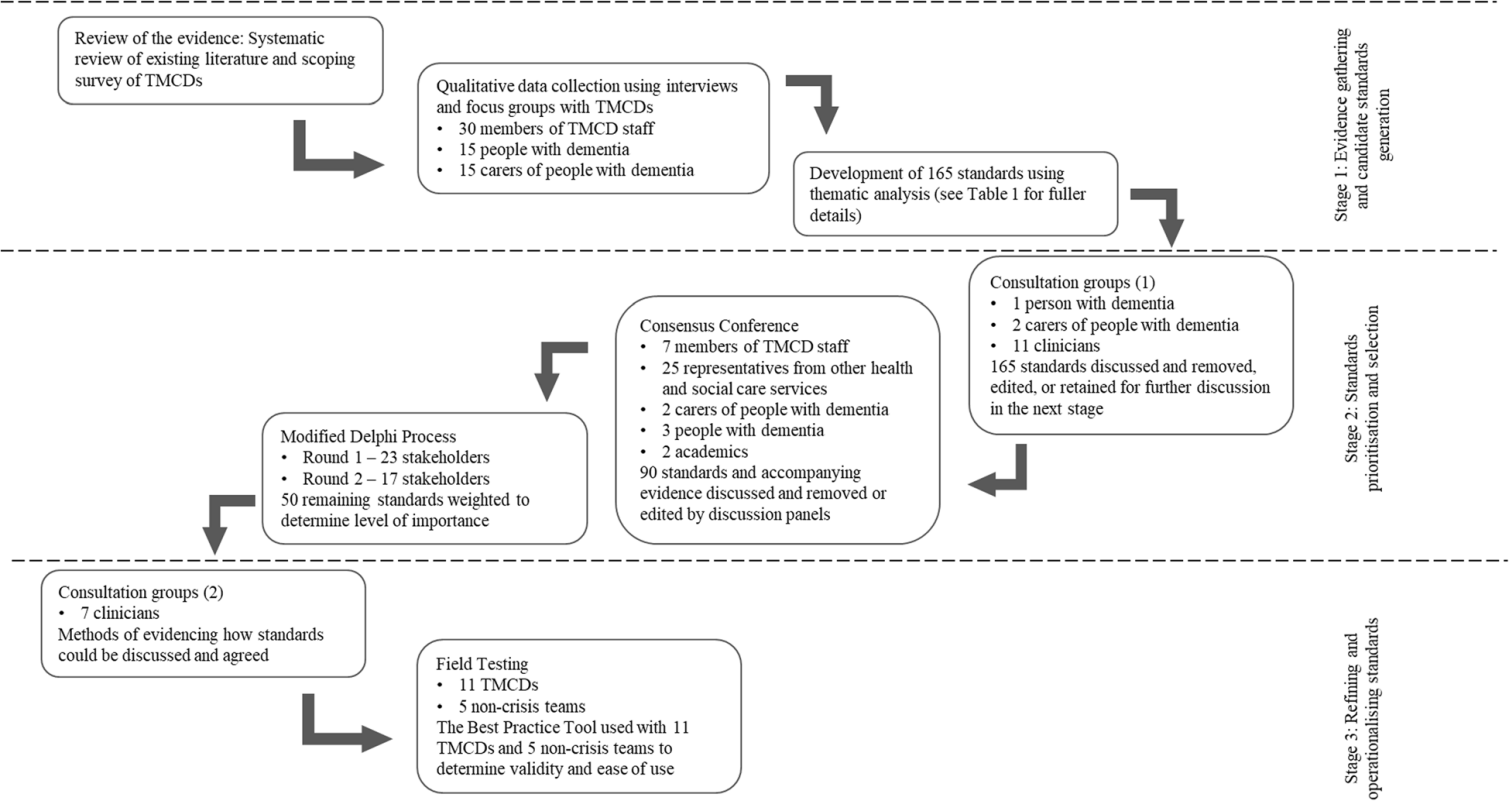
Participant and stakeholder flow though the consensus process

Participants involved in each stage are shown in Fig. 1.

### Procedure

The consensus process had three stages, shown in Fig. 1.

#### Stage 1

Evidence gathering and generation of candidate standards.

### Systematic review and scoping survey

A systematic review and scoping survey of TMCDs in the UK supported the concept that TMCDs are effective and provided a picture of current TMCD service delivery. Results are published elsewhere [5].

### Interviews and focus groups

Sixty interviews and nine focus groups were conducted with nine TMCDs. The sample size was based on the concept of information power [17]. The interviews and focus groups had a broad aim, there was little existing theory regarding this topic, and a cross-case analysis was conducted, so a larger sample size than considered typical was necessary, as these factors reduce potential information power. However, the sample (See Fig. 1) was specific, and the quality of dialogue was high due to researchers having appropriate experience and having undertaken pilot interviews. These factors increased the information power and ensured the sample size was sufficient. The interviews and focus groups explored how the team was set up, the services offered/available, operational factors considered important for effective working, and gathered examples of best practice, (or practice that had not gone as well),. Participants are shown in Fig. 1. Carers and people with dementia were not required to be dyads so that carers of people with more severe dementia, who were themselves unable to participate, were able to participate. TMCDs were selected from a pool of teams that had expressed interest during the scoping survey [5], chosen to be demographically and geographically diverse, reflecting different models of crisis intervention provision. Data were analysed by the first two authors using thematic analysis, following Braun and Clarke’s six stages (see Table 1). The analysis was combined with evidence from the systematic review and scoping survey [5] and used to develop 165 standards that captured the essence of effective TMCD working. Standards were developed by identifying where themes occurred within the data and selecting the component of TMCD service provision being described. These were documented, with new components added each time another was identified, until no further components were found.

**Table 1:** Stages of thematic analysis

Braun and Clarke stages	Our methodology
Familiarising yourself with your data	Data were transcribed verbatim by a transcription company, and quality checked by a researcher (ET). Two researchers (JY and MS) each read half of the whole set of transcripts and noted similarities, contrasting accounts, common patterns, and insights.
Generating initial codes	JY and MS discussed these notes to develop initial codes, paying particular attention to aspects of crisis team practice or service provision that were mentioned, and the outcomes that participants reported as resulting from these activities. This drew on the research team’s existing knowledge from conducting a scoping survey (deductive analysis), but also left space to identify patterns of ideas discussed by the interviewees.
Searching for themes	JY and MS discussed the codes and collated similar codes into potential themes. A theme index of the themes and subthemes was created, where each theme and subtheme was given a numerical identifier.
Reviewing themes	Themes were mapped back on to transcripts in the right hand margin using the theme index numerical identifiers. Every instance of each theme and subtheme was identified and transferred to a framework, which consisted of a matrix for each theme, with a column for each subtheme and a row for each participant. JY and MS checked that all themes remained independent, and any that did not were combined with other themes. Thematic models were discussed with the wider research team as they were developed and refined.
Defining and naming themes	JY and MS used the framework for each theme to summarise the content of each subtheme as a short statement. This enabled the themes and subthemes to be thoroughly operationalised and named accurately, capturing the essence of each theme.
Producing the report	Examples that provided the best and most representative evidence for each theme were highlighted in the framework of each theme. Narrative summaries of the themes were documented and stored for use in further report writing. For the purposes of the consensus process, all aspects of crisis team working and service provision were identified and documented, clustered by similarity or relatedness.

Standards detailing similar or related aspects of practice were grouped together using themes identified to create categories that represented distinct aspects of working practice or service provision, and these were refined throughout the process.

#### Stage 2

Prioritisation and selection of standards.

### Consultation groups (1)

Two consultation groups with 14 stakeholders reduced the number of standards to a manageable amount prior to the consensus conference. Stakeholders were not considered participants, but represented critical friends to the project and were clinicians from TMCDs or other health and social care services, and members of the Patient and Public Involvement (PPI) group. Stakeholders represented a range of disciplines and expertise (e.g. a consultant geriatrician, an occupational therapist, and a person with dementia) and were drawn from local research and health service communities. The groups reviewed each category of standards in turn, facilitated by the research team. Each group reviewed half of the original 165 standards. Stakeholders categorised each standard as: ‘highly important’, ‘moderately important’, or ‘not important’ to TMCD working. Some standards were rated ‘Undecided’ if a decision could not be made and these were prioritised for discussion at the consensus conference. Items deemed highly important by the majority of the group were retained for inclusion in the consensus conference. Items deemed not important by the majority of the group were discarded from further inclusion. Items considered moderately important by the majority of the group were combined with other items or modified. The research team provided contextual evidence from the systematic review, scoping survey, and qualitative work and ensured that standards aligned with the evidence after changes were made. The initial 165 standards were reduced to 95 standards.

### Consensus conference

The consensus conference aimed to further refine and reduce the statements to a Best Practice Model that could be taken forward to field-testing. The consensus conference was a 1 day event involving 39 participants. Participants were selected via local and national research, practice, and PPI communities through contacts developed by the research team during the earlier stages of the research. Participants are shown in Fig. 1. All participants had a working knowledge of crisis in dementia through personal or professional experience and represented expert viewpoints. The process used was similar to a consensus development panel, which involves organised meetings of experts in a given field from a variety of disciplines [16]. Unlike nominal group techniques, the consensus conference approach is not anonymised, nor does it rely on standards having to reach a particular threshold of agreement to be retained. The face-to-face interactive aspect of the consensus conference provides a means to synthesise the best available evidence by encouraging interactions between people, drawing on and expressing multidisciplinary perspectives, with experts taking ownership of material on topics that directly impact them. It is an iterative, systematic, practical approach, enabling consensus to be reached in a day.

Participants were allocated to one of five discussion groups, each with approximately six participants. Groups were facilitated by members of the research team and the PPI group. Participants were allocated to groups on their experience of components of TMCD service delivery contained in each category, and where possible included a person with dementia and a family carer. Prior to the consensus conference all facilitators were trained in facilitation skills to moderate discussions and ensure everyone had equal opportunity to participate. Participants received the standards and their group allocation before the consensus conference. Groups considered one or two categories of standards (depending on the size of the category) but were encouraged to cross reference standards in other categories.

**Fig. 2:**
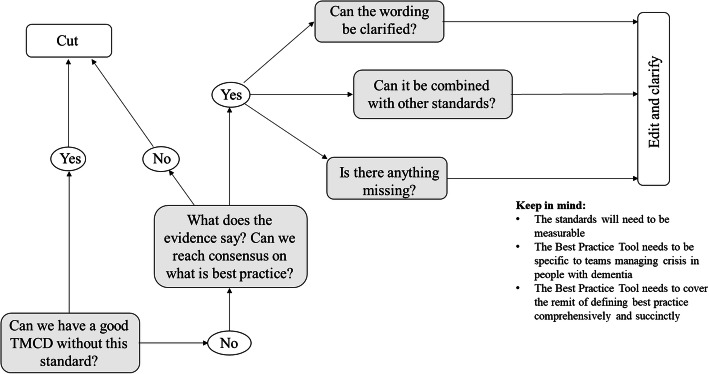
Decision making process used by participants of the consensus conference

Participants received a workbook containing the 95 standards. Each standard was detailed in full and presented with quotes from the qualitative work that provided an evidence-base and contextual background. Presenting data from previous stages of the process enabled decisions to be reliant on the evidence rather than solely the personal experience of an individual. The facilitators of each group guided discussions by following the decision-making process outlined in Fig. 2. Consensus was achieved when the whole group agreed on the inclusion and wording of each standard. Standards not reaching consensus in groups were considered by the whole conference, with further ideas identified until agreement was reached.

The consensus conference reduced the number of standards to 50. The research team reviewed these standards to ensure no duplication or missing elements and that, based on the knowledge gained from the systematic review, scoping survey, and qualitative work, the standards fit with current practice.

### Modified Delphi process

Consensus conference participants acknowledged and agreed that not all standards could be considered equal in their contribution to delivering best practice. This was because some standards underpin others, and consequently standards must be weighted. A modified Delphi approach [16] was used to conduct a points allocation task, where stakeholders were invited to allocate a total of 100 points to the 50 standards by giving each standard a score of 1, 2, 3, or 4. Twenty-three stakeholders participated in round one of the points allocation task. Stakeholders consisted of participants who had attended the consensus conference, stakeholders from the consultation groups, members of our PPI group, and academics and were invited to participate by email. Scores from round one were collated and averaged to produce a score from one to four for each standard. In round two, the points allocation task was returned to the stakeholders with the average score shown next to each standard. Seventeen stakeholders were available to participate in round two, and scored the standards again taking account also of the average score. Scores were again collated, averaged, and allocated to each standard.

#### Stage 3

Refining and operationalising standards.

### Consultation groups (2)

A third consultation group (Fig. 1) to determine the type and availability of evidence required to demonstrate fidelity to the standards, and how this could be collected. Standards developed earlier in the consensus process that were not retained in the final 50 were discussed and refined for use, where relevant, as potential indicators of evidence for each standard.

**Fig. 3:**
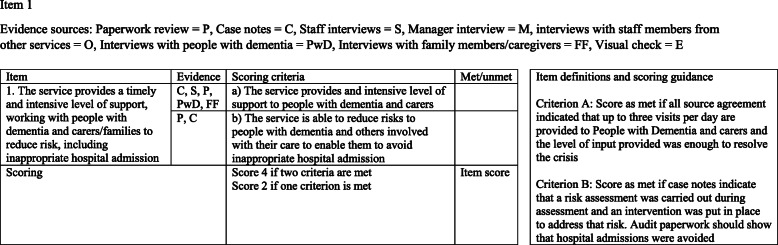
An example of the scoring sheets used in field-testing the Best Practice Tool

The research team created scoring sheets for use when reviewing TMCDs, based on feedback from consultation groups, and drawing inspiration from the CORE study [14], which used a similar review process to the present study (see Fig. 3 for an example).

### Field-testing

The field-testing process was based on the process used in the CORE study [14]. Review days were held with 12 TMCDs and five non-crisis teams. Non-crisis teams included Community Mental Health Teams for older people (CMHT-OPs). Some CMHT-OPs were in areas where a TMCD also operated, and there was little overlap in working practices or service provision between these teams and the local TMCD, but others operated in areas without TMCD provision. Prior to the review day, the research team contacted the TMCD/CMHT-OP to explain the process, and support the teams in making arrangements for the review process. On the review days, a reviewing team comprising two members of the research team, a member of staff from a different TMCD, and a member of the PPI group visited each team with the scoring sheets (Fig. 3). Reviewers began the day with a tour of the team base, and then conducted all other activities to fit with the needs of the team, either in pairs or individually, and some evidence was collected at a later date. The reviewers met to complete the scoring sheet (see Fig. 3) and provided feedback to the team. Draft reports were provided to teams, allowing for clarifications, further evidence to be collected, and any changes to the fidelity score were agreed by the reviewing team. Scores between TMCD and non-crisis teams were compared to determine discriminant validity of the measure, to assess for floor or ceiling effects, and (in conjunction with feedback from team managers) to determine face validity of the Best Practice Model.

## Results

### Qualitative data

Thematic analysis identified 165 standards, which formed 18 categories each capturing an aspect of crisis working for TMCDs. Table 2 displays the categories identified at this stage, with an example from each category, and supporting quotes.

**Table 2:** Original categories, example standards, and supporting quotes, identified from the qualitative work

Category	Example Quote	Original standard
Service purpose	So we get a lot of referrals asking us ‘Please can you just maintain contact’ or ‘Please can you just pop in a keep visiting this person’. As much as we would love to do that, we are not commissioned to do that, and we don’t have the staffing to do it. (Staff 04–02)	Staff members are aware of the aim of the service and can communicate it clearly to other healthcare professionals, service users, and people who support service users (e.g. family carers)
Team values	He kept looking at his watch, you see, and I thought, I know they’ve only got so much time. (Carer - 05-08)	Service users and carers should not feel rushed during face to face contact with service users and carers
Reflexivity	So for about two hours we just talk about what is going on in the team, like how we can improve, like anything wrong that we need to iron out. (Staff 03–02)	Team members are informed of quality improvement of the service, team performance, policies, changes, and development opportunities
Coordination of the service	They always just did exactly what they said they would do (Carer 01–05)	The team is reliable in keeping appointments and then actioning what is agreed
Decision making	I will work out my case load and who is the priority and within my case load I have got at the moment somebody who needs seeing weekly. I will work out with them what they need at that time (Staff 04–03)	Team members are able to make day to day decisions autonomously
Outcomes	It’s really, really hard to quantify a person’s recovery (Staff 02–04)	Outcome measures are appropriate to the service user and carer’s needs and can document their progress whilst in contact with the team
Accessibility of the service	Sometimes most of the feedback we get is ‘you call yourself a crisis team?’, you know when someone is in dire need of help and they call in the office about 9 o’ clock … you just almost wish someone was there (Staff 01–04)	The service is operational during hours that are appropriate to patient needs
Responsiveness of the service	So we sort of put them in terms of their needs to red, amber, green, or inpatient and that would determine the contact we make (Staff 01–03)	The service prioritises service users according to level of risk to themselves or others involved in their care
Staffing the service	Band 6 s would be expected to go and see somebody in their own home because of the risks involved … whereas a band 5 would do this in the care home because there is always people around afterwards (Staff 03–01)	There are clear job roles and boundaries within bandings for team members
Leadership	The good thing about the team here is the manager, one of the managers [manager name] is actually more based, she used to work in older people’s services so she understands older people’s services much better, the needs of people with dementia (Staff 02–03)	The team leader has specialist knowledge in older adults and dementia
Supervision and training	Yes and we ran a training course, me and my colleague here, on safeguarding and procedures and things like that and the Managers attended and the Psychiatrists attended, you know it was kind of, it was and then the Psychiatrists run training on areas that we feel we are lacking as well and so it’s good, exchange is good (Staff 02–04)	Team members have the opportunity to engage in training led by experienced and senior members of the team
Joint working	Some of the referrals aren’t very deep, three or four lines. Some of them are brilliant, they give you loads of information. But others they don’t. It can be a bit frustrating (Staff 04–01)	Crisis teams are explicit with GPs about what information is required in a referral, and what physical health checks must be completed prior to referrals
Team base environment	We hot desk, which is a bit of a nightmare if there’s no computers, but we’ve all got laptops, so you can be sat on your knees sometimes at a little desk in the corner (Staff 02–04)	The crisis team have access to an appropriate space to facilitate MDT meetings, complete paperwork and conduct telephone calls
Referrals	I can’t even make a guess [at referral rates] (Staff 03–02)	Service user flow should be measured for the purposes of service planning and all team members are made aware of this information
Assessments	I didn’t want to do writing. Writing has been a down-turn for me all my life (Service User 01–21)	The purpose and outcomes of assessments conducted by the team should be clearly explained to service users and carers
Psychosocial interventions	Well mostly they would sit and talk to you and just give you tips on how to handle dementia … he would say ‘well, next time why don’t you try this’ or ‘maybe he did that because …’. Do you know what I mean? (Carer 01–05)	The team provides education and support to carers to help them support the service user at home, which may include information about dementia, including basic information about what diagnosis the service user has and what the symptoms may include and signposting to available resources and services for service users and carers where relevant
Pharmacological interventions	Medication reviews, just like is part and parcel of what you would do if you get called out. (Stakeholder Focus Group 01)	The team should review or be able to arrange for a review of medication that the service user is prescribed
Onward referral	And then they would come perhaps a couple of times and then they would say, “well we think everything is ok now, we are going to close the books on you” which is the one thing that I find a bit unacceptable really, because the trouble is, once they have closed the book down on you, you then have to get in touch with your doctor and get the doctor to call them out again (Carer 03–17)	Service users and carers are adequately prepared for discharge from the service, are aware of how to re-access the team if necessary and are involved in the decision to discharge. Written and face-to-face information is offered.

### Consultation groups (1)

The consultation groups reduced the number of standards from 165 to 95. One hundred and fifteen standards were rated as ‘highly important’, 10 ‘moderately important’, 7 undecided and 33 ‘not important’. Examples of standards rated are: highly important ‘*The team uses established and streamlined documentation that is appropriate to team member needs and kept up to date*’; moderately important ‘*Team members should be distinguishable by service users and carers from other health and social care professionals’*; undecided ‘*The team set expectations of the service with service users and carers at the beginning of the service’s involvement with the service use*r’ and not important ‘*The crisis team is co-located with other relevant services*’. Of the 165 statements, 61 were combined with at least one other statement, 25 were retained in their original wording, 19 were modified, 27 prioritised for consideration at the consensus conference and 33 discarded. Standards were re-grouped into five overall categories: (1) management, (2) resources available to support rapid assessment and intervention, (3) assessment, (4) interventions, and (5) onward referral. Categories were based on the original groupings developed from the thematic analysis, but refined to conceptually match the components of TMCD working included in each category.

### Consensus conference

Consensus conference participants reduced the number of standards from 95 to 50. Of the 95 statements, 58 were combined with at least one other statement, 26 were modified, and 11 discarded. The resulting standards represented measurable principles that were either specific to TMCDs, or essential characteristics of high-quality community healthcare teams that underpinned crisis work. For example, a standard specific to TMCDs was: ‘*Service staff work to build a rapport with the person with dementia and their carers/families to ensure they are involved in decision making*’, whereas a standard representing an essential quality was: ‘*All service staff feel confident to contribute to decision making in an open and supported process’*. An example of two standards that were combined and clarified: ‘*Team members have the means to communicate effectively and efficiently within the service*’; and ‘*The team uses established and streamlined documentation that is appropriate to team member needs and kept up to date’*, which became ‘*Service staff have the means to communicate effectively using established documentation that is organised to avoid duplication and is up to date’*. For the aetiology and development of each standard see Supplementary File 1.

A reduction in standards enabled a restructuring of the categories, resulting in (1) the crisis service, (2) rapid assessment and intervention, and (3) service resources. Consensus was also reached on terminology used to refer to people described by the standards, changing from ‘service users’ to ‘people with dementia’, and from ‘teams’ and ‘staff’ to ‘service staff’.

**Table:** 

**The crisis service:** 22 standards relating to services’ purpose, values, procedures, and improvement. **Rapid Assessment and Intervention**: 14 standards relating to accessibility, assessment, and intervention. **Service Resources**: 14 standards relating to staffing, joint working with other services, and the team base.	

### Modified Delphi process

The finalised list of statements and their average scores from both rounds of the Delphi process, and the final agreed scores for each standard are shown in Table 3.

**Table 3:** The finalised standards agreed at the consensus conference with allocated scores following the Delphi process

Standard	Delphi Round 1	Delphi Round 2	Final allocated score
**The Crisis Service**			
The service provides a timely and intensive level of support, working with people with dementia and carers/families to reduce risk, including inappropriate hospital admission.	3.4	3.8	4
The service communicates a clear, flexible definition of crisis and its own aims to other services, people with dementia and their carers/families.	2.0	2.2	2
The service has a definition of when a crisis is resolved to a point where intensive support from the service is no longer required.	1.7	1.5	2
Service operational policies outlining the purpose and eligibility criteria are accessible by service staff.	1.3	1.1	1
The service is person-centred and care is planned to meet the needs of the person with dementia and their carers/families. Service staff are caring, approachable and professional, and treat people with empathy and understanding.	3.3	3.5	4
Service staff work to build a rapport with the person with dementia and their carers/families to ensure they are involved in decision making.	2.3	2.3	2
All service staff feel confident to contribute to decision making in an open and supported process.	1.5	1.3	1
Service staff explain the care to be delivered to the person with dementia and their carers/families at the start and throughout their involvement. Information is timely, accurate and relevant to the needs and wishes of the person with dementia and their carers/families.	2.5	2.4	2
People with dementia and their carers/families have the opportunity to speak with service staff separately and together; they are not rushed during face-to-face contact.	2	1.9	2
Staff are aware of cultural and minority group issues that may affect people with dementia and their carers/families, and know how to enhance their approach to support them.	2	1.9	2
People with dementia and their carers/families have a named worker to support consistency of staff working with them.	2	2.1	2
The service has a system for prioritising risk and assessing required levels of support for people with dementia.	2.8	2.9	3
Each service has a senior qualified ‘duty worker’ (shift coordinator) who allocates work each day and who oversees all calls about patients.	2	1.7	2
Service staff are able to make day-to-day decisions autonomously, in keeping with their levels of experience and in line with their professional competencies where relevant.	1.9	1.9	2
Service staff have the means to communicate effectively using established documentation that is organised to avoid duplication and is up to date.	1.7	1.5	1
A daily handover takes place to communicate information about people with dementia between service staff.	2.1	1.8	2
The service uses a centralised diary system led by the shift coordinator to know where service staff are and availability for new referrals.	1.5	1.3	1
Case load, mix and flow are measured and used to assist the organisation and planning of the service, with the staff working rota allowing for flexibility regarding staff absence and working patterns.	1.6	1.3	1
Service satisfaction information is collected from people with dementia and their carers/families using an appropriate measure. The whole service is aware of how it is evaluated in terms of satisfaction and performance, and how these results are acted upon. The service has a process to manage all feedback.	1.7	1.5	1
Service staff are informed of and involved with quality improvement initiatives, affording the flexibility to think creatively.	1.4	1.3	1
All service staff have regular clinical supervision that is separate from managerial supervision and is in accordance with professional and NHS Trust standards.	2.3	2.3	2
All service staff have regular opportunities for continuing professional development to support clinical and non-clinical skills related to the range of crises that affect older people with dementia.	1.9	1.7	2
The service operates outside normal working hours and signposts to other community-based support when the service is closed outside of these hours.	2.6	2.7	3
The service communicates its referral process to people with dementia, their carers/families, and other relevant organisations.	1.5	1.7	2
**Rapid Assessment and Intervention**			
Following referral, the service makes initial contact on the same day and the person with dementia is seen within the next working day for appropriate crisis referrals.	2.6	2.7	3
At a minimum, the service is accessible by telephone and if an answerphone or voicemail system is used, calls are returned and responded to according to risk.	1.9	2.0	2
Service staff can see the person with dementia at their usual place of residence.	2.2	2.2	2
Service staff use a comprehensive assessment that includes standardised measures where appropriate, risk assessments, and the views of the person with dementia and their carers/families to inform care planning.	2.8	2.8	3
The purpose and outcomes of assessments used by service staff are clearly explained to the person with dementia and their carers/families.	2	1.9	2
Service staff take an holistic approach, considering physical health, mental health, and social needs.	2.7	2.9	3
Service staff provide information and education relevant to the specific dementia diagnosis, tailored to individual needs, to help carers/families support the person with dementia at home.	2.3	2.1	2
Service staff provide interventions to improve quality of life for the person with dementia and their carers/families by providing practical assistance and problem solving techniques.	2.4	2.5	3
Service staff review medication and monitor its effectiveness. Service staff have access to prescription of medication and are able to dispense it.	2.4	2.0	2
Service staff engage in interventions to prevent further crisis; these may include assessment, advice and support for other professionals.	2.3	2.4	2
Service staff signpost and facilitate referrals to other services including respite care.	1.7	1.4	1
People with dementia and their carers/families are involved in the decision to discharge, are adequately prepared for discharge, and are aware how to re-access the service if necessary. Verbal and written information is offered which includes information about onward services organised by the crisis service.	2.2	2.5	3
The service takes a multidisciplinary approach and has awareness of, and immediate access to, other relevant professional disciplines.	2.4	2.6	2
The clinical lead for the service has specialist knowledge and skills relevant to working with older people and with dementia.	2.5	2.3	2
Service staff have specialist dementia knowledge and skills through training and/or appropriate clinical experience.	2.5	2.7	3
The service has administrative support that is sufficient to meet current demand.	1.6	1.6	1
The service has an operational plan which includes staff mix and bandings, and roles and responsibilities.	1.4	1.2	1
Service staff understand all relevant legislation.	1.9	1.7	2
The service is embedded within established pathways of care and policies exist for working with all other relevant agencies, to include social care, emergency services, charities, and the voluntary sector. Other agencies and services have an accurate perception of the crisis service and its remit.	2.4	2.6	3
Agreements are in place to support cross-boundary working across geographical and commissioning areas, for example, with neighbouring health services and local authorities.	1.6	1.4	1
The service liaises with the person with dementia’s General Practitioner (GP). The service is explicit with GPs about what timely information is required in a referral, and what physical health checks should be undertaken prior to referral. The service includes GPs in decision making where relevant and through correspondence.	2.4	2.1	2
The service has good communication with other services involved in the care of the person with dementia and their carers/families to avoid unnecessary duplication of assessments.	1.8	1.6	2
Joint visits between service staff and professionals from other agencies take place when necessary.	1.4	1.2	1
Service staff and professionals from other services attend each other’s meetings when necessary, and appropriate escalation procedures are established and shared when required for complex cases.	1.1	1.0	1
**Service Resources**			
The service has access to appropriate space to facilitate Multi-disciplinary Team (MDT) meetings, and for staff to complete paperwork and conduct telephone calls of a confidential and/or sensitive nature.	2	1.9	2
There is provision of Information Technology (IT) resources and associated IT support appropriate to the needs of the service. This includes access to computer systems, including electronic notes, to enable working remotely from various locations.	1.8	1.7	2

### Field-testing

The Best Practice Tool was piloted with 16 teams (11 TMCDs and five non-crisis teams). Reviews were completed in all teams, and all participating teams received a report and total score. TMCDs tended to score higher than non-crisis teams, suggesting good discriminant validity. Initially, ceiling effects occurred in scoring across both types of teams, therefore changes were made to the criteria required to achieve the maximum scores for each standard in the Best Practice Tool. This made it more difficult for teams to achieve high scores, resulting in greater variation in scores across participating teams at this field-testing stage, allowing for a more nuanced exploration of areas for improvement. Scoring changes were documented and implemented after ten teams (seven TMCD and three non-crisis) were reviewed. These teams were re-scored with updated criteria, with the final six using updated criteria only. Scores ranged from 48 (non-crisis team) to 92 (TMCD) within a possible range of 0–100. The median score for all 17 TMCDs using the revised scoring criteria was 74.5, ranging from 67 to 92, and the median score for non-crisis teams was 60, ranging from 48 to 72. Scoring for one non-crisis team was incomplete due to data collection issues. Feedback from managers suggested good face validity, in that the Best Practice Tool captured practice in a realistic way, and managers generally found the process useful, non-threatening, and appreciated identification of areas for improvement. In particular, teams who provided a staff member to be a reviewer for another team found the experience positive, commenting that it was helpful to see how other teams operate and create networks with other professionals in similar services. The review days were, however, lengthy, and required prior preparation, which was burdensome upon busy services.

## Discussion

### Main findings

This study has identified a model of best practice for TMCDs that represents the very essence of crisis care for people with dementia, and is a product of an objective consensus process involving stakeholders who are experts by experience, qualification, or professional training. The Best Practice Model provides a clear role and method of working for TMCDs, emphasising that TMCD service provision is necessary to meet the needs of people with dementia and their carers who experience crisis situations. Field-testing the Best Practice Tool with TMCDs and non-crisis teams demonstrated that it can be successfully implemented, and can distinguish TMCDs from non-crisis teams, provide helpful feedback, celebrate areas of good practice, and identify areas for service improvement.

The process used here highlights the utility of consensus methodology in establishing agreement on topics with limited empirical evidence and showed that the process can be conducted rigorously [16]. The stages involved ensured that equal voice was given to different groups of stakeholders, including people with dementia and TMCD practitioners, and that the model developed was realistic in the context of current service provision.

### Limitations

This research and the resultant Best Practice Model focuses on services provided in England, UK. It is likely that our findings will apply to the devolved nations of the UK due to similar health and care structures, but applicability beyond the UK is unknown. The conceptualisation of crisis for people with dementia internationally is under-researched, but definitions of crisis and service organisation seem similar [18]. Important differences in service provision internationally are likely to arise from differing demographics and geographies, for example in rural Australia where assessment teams can only visit community-dwelling older people infrequently [19]. This research provides a starting point for planners in other countries to build upon existing services or develop new services that meet the needs of people with dementia who experience a crisis.

During the process, it was clear that being overly prescriptive would be counter-productive, as best practice is often dependent on local context and factors such as case-mix, which vary according to local demographics. This approach sacrifices precisely defined standards, for which a high degree of reliability could be calculated, in favour of standards that have an enhanced contextual validity and reflect the need for warranted variation that enables patient-centred care. Consequently, some standards are quite general. However, these standards have captured crisis team working, since non-crisis teams tended to score lower in the field-testing of the Best Practice Tool.

The psychometric properties of the Best Practice Tool were not identified during this process and will be established in future work. The number of teams involved in the field-testing was too small for meaningful statistical comparisons to be conducted. Test-retest reliability could not be established given the substantial burden of performing a review day for both participating teams and reviewers, a finding consistent with conclusions drawn by the CORE study [14]. Similarly, inter-rater reliability could not be established due to the large number of raters involved, but this calculation would be desirable in future research. No single reviewer looked at every piece of evidence gathered, as reviewers completed separate tasks in pairs during the day and the score was a product of discussion, rather than individual decision making. Training and the agreement of a score by the whole reviewing team aimed to enhance scoring reliability. Not all information was necessarily present on the review day, and consequently the score received by the team may be lower than expected. However, TMCDs could challenge provisional scores and provide additional evidence if available. These features of the process, and involvement of a consultation group to determine the type and availability of evidence that teams could access, further enhanced reliability of the Best Practice Tool, and demonstrated that the tool can be usefully applied as a self-assessment tool in future, where reviewers are likely to be different each time. The criterion validity of the tool should be explored, since it is unknown how well a score on the tool relates to clinical outcomes for people with dementia and carers.

### Strengths

A key strength is the involvement of people with dementia and their carers, and TMCD practitioners. People with dementia and their carers formed our PPI group and not only advised on how the research was conducted, but also played a vital role in research delivery. PPI members collected data, co-facilitated consensus discussion groups, and were members of reviewing teams. People with dementia, their carers, and clinicians were involved as critical friends in the consultation groups and modified Delphi process. The involvement of people who will use the Best Practice Tool in their clinical practice, and of people using health and care services, ensured that the Best Practice Tool is realistic and achievable.

### Clinical implications

Using the Best Practice Tool as a self-assessment exercise is feasible and the Tool can highlight areas for service improvement. The US EBP project [20] used a similar fidelity process, which allowed services to identify their strengths and areas for service improvement, and did improve quality of service provision. This suggests the standards developed here can be used by TMCDs to improve practice. As a by-product of the process, clinicians visited other teams and shared good practice, suggesting opportunities for a community of practice across TMCDs. Using the Best Practice Model and Tool, and sharing knowledge, could spread innovation and ideas, and provide opportunities for standardisation of good practice nationally. The standards could provide national-level benchmarking data about practice and variation in TMCD services, information which is useful to policy makers and service planners. The CORE adult crisis team fidelity scale provides an example of this as it was used in a national survey [21], and is recommended in policy [22].

Crisis teams specifically for people with dementia do not appear to be standard practice at a nationally or internationally and appear rarely in research. A systematic review identified crisis resolution or home treatment teams not specific to dementia exist in other countries [23], and studies have evaluated similar hospital at home concepts for mental health support in Australia [24], France [25], and Spain [26], although these are not specific to dementia crisis. Recommendations by Alzheimer Europe encourage development of services which operate with a flexible approach and react to crisis situations at home in a timely and immediate fashion [27]. However, this distinct function makes other elements of best practice, such as continuity of staff, challenging [28] and these issues should be considered when designing services. The Best Practice Model developed here, and the process used to develop it, will be of interest internationally to planners who could benefit from establishing TMCDs as a model of working.

### Research implications

Psychometric testing is needed to quantify the inter-rater reliability, and criterion validity of the Best Practice Tool to ensure scores achieved relate to measurable outcomes for TMCDs. This aspect of the work forms part of a larger programme of research to develop a Best Practice Toolkit to support TMCDs in providing high-quality and effective care. The areas for service improvement highlighted through field-testing the Best Practice Tool will be used to identify strategies and resources for quality improvement. The resulting Toolkit developed from this work will be evaluated alongside the Best Practice Model in an RCT.

## Conclusion

This article describes the development of a Best Practice Model for services that provide support to people with dementia who experience a crisis. Key aspects of TMCD working that distinguish them from non-crisis services are the high intensity, short duration support, and rapid response. TMCDs assess and establish on-going needs, and broker support from other services to meet needs long term. Whilst these standards focus on TMCDs in England and require further psychometric testing, their broad evidence-base and non-prescriptive nature make this Best Practice Model and Tool ideal for use in TMCD practice, service planning, service improvement and national benchmarking.

## Supplementary information

**Additional file 1.**

## Data Availability

Most data generated or analysed during this study are included in this published article, its supplementary information files or in other published work. The complete data for the qualitative data analysis is available on reasonable request from the corresponding author.

## Abbreviations

AQUEDUCT: Achieving Quality and Effectiveness in Dementia Using Crisis Teams
CCQI: Centre for Quality Improvement
CMHT-OP: Community Mental Health Teams for Older People
CORE: Crisis Resolution Team Optimisation and Relapse Prevention
CRT: Crisis Resolution Team
RCP: Royal College of Psychiatrists
TMCD: Teams Managing Crisis in Dementia
UK: United Kingdom
