# Supplementary material for: Developing a model of best practice for teams managing crisis in people with dementia: a consensus approach
Source: BMC Psychiatry. 2020 Oct 13;20:505. doi: 10.1186/s12888-020-02899-0 (PMC7552369; doi:10.1186/s12888-020-02899-0)
Supplement: Supplementary file 1 — Additional file 1. [file 12888_2020_2899_MOESM1_ESM.docx]

*Supplementary file 1*: Candidate statements, supporting evidence and their journey throughout the consensus process

Key: SI=staff interviews, CI=carer interviews, SUI= service using interviews, FG= Focus Groups, S=Survey, SR=Systematic Review

H=Highly Important, M=Moderately Important, N=Not Important, U=Undecided and referred to Consensus Workshop

| **No.** | **Standard** | **Evidence** | **Consultation group outcome** | **Action** | **Consensus workshop Action** | **Consensus Conference Outcome** |
| --- | --- | --- | --- | --- | --- | --- |
|  | **Management – Service Purpose** | | | | | |
| 1 | The service has a clearly defined aim | SI, SUI, CI | H | Combined with standard no. 4 |  |  |
| 2 | Staff members are aware of the aim of the service and can communicate it clearly to other healthcare professionals, service users, and people who support service users (e.g. family carers) | SI, SUI, CI | H | Retained | Combined with no. 4 |  |
| 3 | The service is able to reduce risks to service users and others involved with their care to enable them to avoid inappropriate hospital admission | SI, S, SR | H | Combined with standard no. 6 |  |  |
| 4 | The service has a clear, yet flexible, definition of what circumstances constitute a crisis that is shared with other services, service users and families, based on a theoretical model | SI, SUI, CI | H | Combined with standard no. 1 and modified: The service has a clear, yet flexible, definition of what circumstances constitute a crisis that is shared with other services, service users and families | Combined with no. 2 and modified | The service communicates a clear, flexible definition of crisis and its own aims to other services, people with dementia and their carers/families. |
| 5 | The service has a definition of when a crisis is resolved | SI, SUI, CI, S | H | Modified: The service has a definition of when a crisis is resolved to a point where intensive support is no longer required | Modified | The service has a definition of when a crisis is resolved to a point where intensive support from the service is no longer required. |
| 6 | The team provides an intensive level of support to service users and carers. The level of intensity should reduce as the crisis resolves | SI, FG, S | H | Combined with standard no. 3 and modified: The team provides an intensive level of support to service users and carers to reduce risks to service users and others involved with their care to enable them to avoid inappropriate hospital admission | Combined with no. 9 and modified | The service provides a timely and intensive level of support, working with people with dementia and carers/families to reduce risk, including inappropriate hospital admission. |
| 7 | The team is a gatekeeper of inpatient beds by preventing hospital admissions where possible and admitting service users only when it is in their best interests | SI, FG | N | Discarded |  |  |
| 8 | The team is a gatekeeper of inpatient beds by preventing service users from being sectioned when an alternative can be provided in the community setting | SI, FG | N | Discarded |  |  |
| 9 | The team provides immediate support to service users and carers | SI | H | Retained | Combined with no. 6 |  |
| 10 | Policies underpinning the purpose of the service exist and are easily accessible by all team members | SI | H | Combined with standard no. 11  Policies underpinning the purpose of the service exist and are easily accessible by all team members. All team members know the eligibility criteria for the service. | Modified | Service operational policies outlining the purpose and eligibility criteria are accessible by service staff. |
| 11 | All team members know the eligibility criteria for the service | SI, S | H | Combined with standard no. 10 |  |  |
|  | **Management – Team values** | | | | | |
| 12 | The service is patient-centred and care is planned to meet the needs of each service user and their families | SI, SUI, CI, FG, S | H | Retained | Combined with no. 24 and modified | The service is person-centred and care is planned to meet the needs of the person with dementia and their carers/families. Service staff are caring, approachable and professional, and treat people with empathy and understanding. |
| 13 | Service users are involved in decision making unless they are unable to participate due to a lack of mental capacity | SI, SUI, CI | H | Combined with standard no. 14 |  |  |
| 14 | Formal and informal carers, family members, and important people in the service user’s life are involved in decisions and care planning | SI, SUI, CI | H | Combined with standard no. 13  Service users are involved in decision making unless they are unable to participate due to a lack of mental capacity. Formal and informal carers, family members, and important people in the service user’s life are also involved in decisions and care planning. | Combined with no. 20 | Service staff work to build a rapport with the person with dementia and their carers/families to ensure they are involved in decision making. |
| 15 | The team set expectations of the service with service users and carers at the beginning of the service’s involvement with the service user | SUI, CI, FG | M | Retained | Combined with no. 23 and 126 and modified | Service staff explain the care to be delivered to the person with dementia and their carers/families at the start and throughout their involvement. Information is timely, accurate and relevant to the needs and wishes of the person with dementia and their carers/families. |
| 16 | The service uses a positive risk taking approach where service users are supported to take manageable risks that result in beneficial experiences | SI | N | Discarded |  |  |
| 17 | Service users and families have copies of the care plan and other relevant documentation | SI, SUI, CI | H | Combined with standard no. 23 |  |  |
| 18 | Service users and carers have opportunity to talk to team members both separately and as a dyad | SUI, CI | U | Prioritised for discussion at consensus workshop | Combined with no. 27 and modified | People with dementia and their carers/families have the opportunity to speak with service staff separately and together; they are not rushed during face-to-face contact |
| 19 | The team puts the best interests of the person with dementia first | SUI, CI | M | Prioritised for discussion at consensus workshop | Discarded |  |
| 20 | The team members are able to build rapport with the service user and carer | SUI, CI | H | Combined with standard no. 26 The team members are able to build rapport with the service user and carer. Team members should be able to explain things clearly without being patronising. | Combined with no. 14 |  |
| 21 | Team members are able to challenge the views of service users and family carers if there is evidence that they are not in the best interest of the service user | SUI, CI | M | Prioritised for discussion at consensus workshop | Discarded |  |
| 22 | The team should ensure that they engage with the service user as well as family carers | SUI, CI | N | Discarded |  |  |
| 23 | The method of provision of information should be appropriate to the needs and wishes of the service user and carer, by delivering relevant information at an appropriate time | SUI, CI | H | Retained | Combined with no. 15 and 126 |  |
| 24 | Team members should be caring, approachable and professional | SUI, CI | H | Combined with standard no. 30: Team members should be caring, approachable and professional and treat people with empathy and understanding. | Combined with no. 12 |  |
| 25 | Team members are honest about the nature of the crisis with service users and carers | SUI, CI | M | Prioritised for discussion at consensus workshop | Combined with no. 15 |  |
| 26 | Team members should be able to explain things clearly without being patronising | SUI, CI | H | Combined with standard no. 20 |  |  |
| 27 | Team members shouldn’t feel rushed during face to face contact with service users | SUI, CI | H | Modified: Service users and carers should not feel rushed during face to face contact with service users and carers | Combined with no. 18 |  |
| 28 | Team members feel confident to challenge each other when making decisions in an open and supportive manner | SI | H | Modified: Team members feel confident contribute to the decision making process in an open and supportive manner | Modified | All service staff feel confident to contribute to decision making in an open and supported process. |
| 29 | Staff are aware of cultural issues that may affect service users and carers, and how to enhance their approach to support people from minority groups | SI | H | Retained | Modified | Staff are aware of cultural and minority group issues that may affect people with dementia and their carers/families, and know how to enhance their approach to support them. |
| 30 | Team members should treat service users and carers with empathy and understanding | SI | H | Combined with standard no. 24 |  |  |
| 31 | Team members are motivated and passionate about working with people with dementia | FG | N | Discarded |  |  |
|  | **Management - Reflexivity** | | | | | |
| 32 | Quality improvement of the service, team performance, policies, changes and development opportunities are discussed in business meetings | SI | H | Modified: Team members are informed of quality improvement of the service, team performance, policies, changes, and development opportunities | Combined with no. 37 and 39 and modified | Service staff are informed of and involved with quality improvement initiatives, affording the flexibility to think creatively |
| 33 | Service user and carer satisfaction with the service is collected using an appropriate measure and evaluated | SI | H | Combined with standard no. 34 and 35:  Service user and carer satisfaction with the service is collected using an appropriate measure and evaluated. The whole team is aware of how the service is evaluated in terms of satisfaction and performance, and how the results are acted upon. The crisis team has a process to manage negative feedback. | Modified | Service satisfaction information is collected from people with dementia and their carers/families using an appropriate measure. The whole service is aware of how it is evaluated in terms of satisfaction and performance, and how these results are acted upon. The service has a process to manage all feedback. |
| 34 | The whole team is aware of how the service is evaluated in terms of satisfaction and performance, and how the results are acted upon. | SI | H | Combined with standard no. 33 and 35 |  |  |
| 35 | The crisis team has a process to manage negative feedback. | SI | H | Combined with standard no. 33 and 34 |  |  |
| 36 | All staff in the crisis team are empowered to make changes. | SI | N | Discarded |  |  |
| 37 | The team has an ethos of supporting new ideas. | SI | H | Combined with standard no. 38  The team has an ethos of supporting new ideas .The service encourages an innovative approach towards problem solving and has the flexibility to think creatively | Combined with no. 32 and 39 |  |
| 38 | The service encourages an innovative approach towards problem solving and has the flexibility to think creatively | SI, SUI, CI | H | Combined with standard no. 37 |  |  |
| 39 | There are opportunities for all team members to be involved in audit activities | SI | H | Prioritised for discussion at consensus workshop | Combined with no. 32 and 37 |  |
| 40 | Change management procedures are documented, are sensitive to the team, and are led by managers | SI | N | Discarded |  |  |
| 41 | There is provision of support for staff members e.g. mindfulness training | FG | N | Discarded |  |  |
|  | **Management – Coordination of the service** | | | | | |
| 42 | The crisis team use a shift pattern that ensures core hours of operation are covered with staffing levels appropriate to the demands of the service. The shift rota allows for flexibility regarding staff absence/working patterns | SI, FG, SR, S | H | Modified: The working rota allows for flexibility regarding staff absence/working patterns | Combined with no. 87 and 139 and modified | Case load, mix and flow are measured and used to assist the organisation and planning of the service, with the staff working rota allowing for flexibility regarding staff absence and working patterns. |
| 43 | Service users and carers have a named individual within the crisis team who is responsible for coordinating their care. This may be known as a named nurse, keyworker or other similar role | SI, FG | H | Prioritised for discussion at consensus workshop | Combined with no. 45 and modified | People with dementia and their carers/families have a named worker to support consistency of staff working with them. |
| 44 | A shift coordinator is responsible for daily coordination of the crisis team. This may involve operating a duty system, being responsive to incoming urgent crises, or allocating new referrals to other members of qualified staff | SI, FG | H | Modified: The shift is coordinated to allow for being responsive to incoming urgent crises, or allocation of new referrals to other members of qualified staff. | Modified | Each service has a senior qualified ‘duty worker’ (shift coordinator) who allocates work each day and who oversees all calls about patients |
| 45 | There should be consistency of staff members for service users | SI, SUI, CI, FG | H | Prioritised for discussion at consensus workshop | Combined with no. 43 |  |
| 46 | The team has system for differentiating between patients requiring levels of support e.g. red/amber/green rating and has autonomy over how patients are categorised | SI, FG, S | H | Retained | Combined with no. 73 and modified | The service has a system for prioritising risk and assessing required levels of support for people with dementia. |
| 47 | Patients are allocated to staff based on staff skills expertise and experience, and likelihood of building rapport | SI | N | Discarded |  |  |
| 48 | Team members have control over their diary and how they plan their day | SI | M | Prioritised for discussion at consensus workshop | Combined with no. 58 and modified | Service staff are able to make day-to-day decisions autonomously, in keeping with their levels of experience and in line with their professional competencies where relevant. |
| 49 | The service uses a centralised diary system to know where team members are and what availability they have to see new patients | SI | M | Prioritised for discussion at consensus workshop | Modified | The service uses a centralised diary system led by the shift coordinator to know where service staff are and availability for new referrals. |
| 50 | A handover takes place daily to communicate information about patients between team members and allocate tasks for the day | SI | H | Modified: A handover takes place daily to communicate information about patients between team members. | Modified | A daily handover takes place to communicate information about people with dementia between service staff. |
| 51 | The team is reliable in keeping appointments and putting agreed plans into action | SUI, CI | H | Modified: The team is reliable in keeping appointments and then actioning what is agreed. | Discarded |  |
| 52 | The service attends a business meeting that is held at least monthly. Where only the manager is able to attend, information from the meeting should be delivered to the team at team meetings. | SI | N | Discarded |  |  |
| 53 | Team members are able to communicate effectively and efficiently within the service | SI, FG | H | Modified: Team members have the means to communicate effectively and efficiently within the service | Combined with no. 55 and modified | Service staff have the means to communicate effectively using established documentation that is organised to avoid duplication and is up to date. |
| 54 | The service uses an electronic case note system | SI | N | Discarded |  |  |
| 55 | The team uses established and streamlined documentation that is appropriate to team member needs and kept up to date | SI | H | Retained | Combined with no. 53 |  |
| 56 | The team uses appropriate methods of documenting team activity | SI | N | Discarded |  |  |
| 57 | Staff members not previously involved in the care of a service user are introduced to the service user by an existing team member | FG | N | Discarded |  |  |
|  | **Management – Decision Making** | | | | | |
| 58 | Team members are able to make day to day decisions autonomously | SI | H | Prioritised for discussion at consensus workshop | Combined with no. 48 |  |
| 59 | A whole team approach is taken with complex cases or risky decisions | SI, FG | N | Discarded |  |  |
| 60 | Decisions that affect the whole team are taken collectively as a team | SI | N | Discarded |  |  |
| 61 | Triage of service users, allocation of cases, and management of cases is decided collectively as a whole team | SI, S | N | Discarded |  |  |
| 62 | The team has control over their budget and how it may be spent | SI | N | Discarded |  |  |
| 63 | All team members are aware of how their service is funded | SI | N | Discarded |  |  |
|  | **Management - Outcomes** | | | | | |
| 64 | The service uses standardised clinical outcome measures | SI, S | N | Discarded |  |  |
| 65 | Outcome measures are appropriate to the service user and carer’s needs and can document their progress whilst in contact with the team | SI, FG | H | Prioritised for discussion at consensus workshop | Combined with no. 141 and 145 and modified | Service staff use a comprehensive assessment that includes standardised measures where appropriate, risk assessments, and the views of the person with dementia and their carers/families to inform care planning. |
|  | **Resources – Accessibility of the Service** | | | | | |
| 66 | Service users are seen at their usual place of residence | SI, SUI, CI, FG | H | Retained | Combined with no. 144 and modified | Service staff can see the person with dementia at their usual place of residence. |
| 67 | The service is operational during hours that are appropriate to patient needs | SI, SR, S | H | Prioritised for discussion at consensus workshop | Modified | The service operates outside normal working hours and signposts to other community-based support when the service is closed outside of these hours. |
| 68 | The team is accessible for referral by other health care professionals in both health, social, and voluntary organisations | FG, S | H | Combined with standard no. 69 and 70: The team is accessible for referral by other health care professionals in both health, social, and voluntary organisations. Service users and carers can refer themselves to the team. People making referrals to the team can access the team directly | Combined with no. 72 and modified | The service communicates its referral process to people with dementia, their carers/families, and other relevant organisations. |
| 69 | Service users and carers can refer themselves to the team | FG, S | H | Combined with standard no 68 and 70 |  |  |
| 70 | People making referrals to the team can access the team directly | FG | H | Combined with standard 68 and 69 |  |  |
| 71 | The team is accessible by telephone. If an answerphone or voicemail system is used by the team, messages are returned within one hour during operational hours. | SUI, CI, FG | H | Prioritised for discussion at consensus workshop | Modified | At a minimum, the service is accessible by telephone and if an answerphone or voicemail system is used, calls are returned and responded to according to risk. |
| 72 | Service users and carers are aware of the team’s existence and remit | SUI, CI, FG | H | Modified: Service users, carers and other relevant organisations are aware of the team’s existence and remit | Combined with no. 68 |  |
|  | **Resources – Responsiveness of the service** | | | | | |
| 73 | The service prioritises service users according to level of risk to themselves or others involved in their care | SI, S | H | Prioritised for discussion at consensus workshop | Combined with no. 46 |  |
| 74 | Service users deemed to be at high risk are seen within four hours of referral | SI | H | Prioritised for discussion at consensus workshop | Combined with no. 75, 76 and 77 and modified | Following referral, the service makes initial contact on the same day and the person with dementia is seen within the next working day for appropriate crisis referrals. |
| 75 | Service users deemed to be at high risk are seen in person, by a qualified staff member, in their own home | SI | H | Prioritised for discussion at consensus workshop | Combined with no. 74, 76 and 77 |  |
| 76 | Service users deemed to be at medium risk are seen within 24 hours | SI | H | Prioritised for discussion at consensus workshop | Combined with no. 74, 75 and 77 |  |
| 77 | Service users deemed to be at low risk are contacted by telephone within 24 hours and seen in person within 72 hours | SI | H | Prioritised for discussion at consensus workshop | Combined with no. 74, 75 and 76 |  |
|  | **Resources – Staffing of the service** | | | | | |
| 78 | The service includes a multidisciplinary team. This includes but is not limited to the following professions: Nurses, Consultants, Psychologists, Occupational Therapists, Social Workers, Physiotherapists, Speech and Language Therapists, support workers | SI | H | Modified and combined with standard no. 81, 84, 93 and 95: The service takes a multidisciplinary approach and has awareness of, and immediate access to other relevant disciplines. This could include but is not limited to the following professions: Nurses, Consultants, Psychologists, Occupational Therapists, Social Workers, Physiotherapists, Speech and Language Therapists, Pharmacy and Support Workers | Modified | The service takes a multidisciplinary approach and has awareness of, and immediate access to, other relevant professional disciplines |
| 79 | The service can access medical input from a consultant psychiatrist | SI | N | Discarded |  |  |
| 80 | There is a consultant psychiatrist based in the team | SI | N | Discarded |  |  |
| 81 | The service can access a pharmacist | SI | H | Combined with standard no. 78, 84, 93 and 95 |  |  |
| 82 | The team includes administrative support that is sufficient to meet current demand | SI | H | Retained | Modified | The service has administrative support that is sufficient to meet current demand. |
| 83 | Team members feel valued in their professional identity | SI, FG | U | Prioritised for discussion at consensus workshop | Discarded |  |
| 84 | The team is cohesive and staff are aware of other team members and can name what disciplines are involved | SI | M | Combined with standard no. 78, 81, 93 and 95 |  |  |
| 85 | Team members who have additional skills beyond their clinical expertise e.g. ability to chair meetings or co-ordinate the shift should be acknowledged | SI, SUI, CI | N | Discarded |  |  |
| 86 | There are clear job roles and boundaries within bandings for team members | SI | H | Prioritised for discussion at consensus workshop | Modified | The service has an operational plan which includes staff mix and bandings, and roles and responsibilities. |
| 87 | The service should have a clear plan and contingencies for when staff across different disciplines or bandings are absent. | SI | H | Combined with standard no. 88: The service should have a clear plan and contingencies for when staff across different disciplines or bandings are absent. The service should operate with an appropriate caseload to staff ratio, and vacant positions within the team should be filled at the earliest opportunity. | Combined with no. 42 and 137 |  |
| 88 | The service should operate with an appropriate caseload to staff ratio, and vacant positions within the team should be filled at the earliest opportunity | SI | H | Combined with standard no. 87 |  |  |
| 89 | Team members should possess ‘soft skills’ needed to build relationships with service users and carers, such as friendliness, warmth, and understanding | SUI, CI, FG | N | Discarded |  |  |
| 90 | Team members should be distinguishable by service users and carers from other health and social care professionals | SUI, CI, FG | U | Prioritised for discussion at consensus workshop | Discarded |  |
| 91 | Team members work well together, complement each other’s roles, experience, and responsibilities, support one another, and acknowledge each other’s contributions to the service | SI | N | Discarded |  |  |
| 92 | Team members understand legislation regarding mental capacity | SUI, CI | H | Retained | Modified | Service staff understand all relevant legislation. |
| 93 | Team can access other professional disciplines who are not part of the team for clinical input such as psychology | SI | H | Combined with standard no. 78, 81, 84 and 95 |  |  |
| 94 | Team members have specialist dementia knowledge and skills through training or experience | SI, FG | H | Modified: Team members have specialist dementia knowledge and skills through training or appropriate clinical experience | Modified | Service staff have specialist dementia knowledge and skills through training and/or appropriate clinical experience. |
| 95 | The team includes staff with expertise in physical health | FG | H | Combined with standard no. 78, 81, 84 and 93 |  |  |
| 96 | There is a dedicated member of staff in the team to support carers | FG | U | Prioritised for discussion at consensus workshop | Discarded |  |
|  | **Resources - Leadership** | | | | | |
| 97 | The service lead has specialist knowledge in older adults and dementia | SI | H | Modified: The team leader has specialist knowledge in older adults and dementia | Modified | The clinical lead for the service has specialist knowledge and skills relevant to working with older people and with dementia. |
| 98 | The team leader should be flexible, proactive, approachable, open to ideas and supportive of team members | SI | U | Prioritised for discussion at consensus workshop | Discarded |  |
| 99 | Team leaders should facilitate two way communication between the team and senior management | SI | U | Prioritised for discussion at consensus workshop | Discarded |  |
|  | **Resources – Supervision and Training** | | | | | |
| 100 | Team members have the opportunity to engage in training led by experienced and senior members of the team | SI | M | Retained | Combined with no. 102, 105 and 107 and modified | All service staff have regular opportunities for continuing professional development to support clinical and non-clinical skills related to the range of crises that affect older people with dementia. |
| 101 | There are opportunities for peer supervision between team members of the same level | SI | H | Combined with standard no. 103 and 104 |  |  |
| 102 | All staff have completed training to enhance dementia awareness | SI | H | Retained but suggested it could be combined with other training standards | Combined with no. 100, 105 and 107 |  |
| 103 | All staff are supervised by a member of staff senior to their level, of their choice, on a monthly basis | SI | H | Combined with standard no. 101 and 104 |  |  |
| 104 | Supervision is protected time that is documented by the supervisor and supervisee, and the record is checked by the service lead | SI | H | Combined with standard no. 101 and 103: There are opportunities for supervision in accordance with professional and Trust standards, which should be protected time that is documented and checked | Combined with standard 108 and modified | All service staff have regular clinical supervision that is separate from managerial supervision and is in accordance with professional and NHS Trust standards. |
| 105 | All staff have access to relevant training | SI | H | Retained but suggested it could be combined with other training standards | Combined with no. 100, 102 and 107 |  |
| 106 | All staff have time to take part in reflective practice | SI | H | Combined with standard no. 108 |  |  |
| 107 | All staff have access to training to support non-clinical skills e.g. IT training | FG | H | Retained but suggested it could be combined with other training standards | Combined with no. 100, 102 and 105 |  |
| 108 | Managerial and clinical supervision should be held separately | SI | H | Combined with standard no. 106: All staff have time to take part in reflective clinical supervision, which should be separate from managerial supervision and its occurrence should be documented | Combined with no. 104 |  |
|  | **Resources – Joint Working** | | | | | |
| 109 | The crisis team is embedded within a wider pathway of care and has links to other NHS services (e.g. liaison psychiatry), acute care, and external agencies such as social services | SI, SUI, CI, FG | H | Combined with standard no. 111, 112 and 113: The crisis team is embedded within a wider pathway of care and has links to other NHS services (e.g. liaison psychiatry), acute care, and external agencies such as social services and members of the team are aware of their remit and where they sit within the pathway | Combined with no. 128 and modified | The service is embedded within established pathways of care and policies exist for working with all other relevant agencies, to include social care, emergency services, charities, and the voluntary sector. Other agencies and services have an accurate perception of the crisis service and its remit. |
| 110 | The crisis team has a working relationship with social services | SI, FG | N | Discarded |  |  |
| 111 | The team is aware of the wider pathway to which they belong and are able to communicate this to the service user and carer | SI, SUI, CI | H | Combined with standard no. 109, 112 and 113 |  |  |
| 112 | The team know what the role of other teams operating in the pathway is | SUI, CI | H | Combined with standard no. 109, 111 and 112 |  |  |
| 113 | The crisis team is co-located with other relevant services | SI, FG | N | Discarded |  |  |
| 114 | Professionals from other services involved in current cases are invited to the crisis team MDT meetings | FG | H | Combined with standard no. 115 |  |  |
| 115 | Crisis team members are invited to MDT meetings held by other agencies when joint working is taking place | FG | H | Combined with standard no. 114: TMCD staff members and professionals from other services attend each other’s meetings when necessary and appropriate escalation procedures are established for complex cases | Modified | Service staff and professionals from other services attend each other’s meetings when necessary, and appropriate escalation procedures are established and shared when required for complex cases. |
| 116 | Electronic case-note systems are accessible by all agencies involved in care | SI, FG | N | Discarded |  |  |
| 117 | Other agencies and services have an accurate perception of the crisis team and its remit | SI, FG | H | Combined with standard no. 124 and 128 |  |  |
| 118 | The crisis team is able to engage in a two-way dialogue with commissioners | FG | H | Modified: The crisis team engages in a two-way dialogue with commissioners | Discarded |  |
| 119 | Agreements are in place to support cross-boundary working with neighbouring health services or local authority teams | SI | H | Modified: Agreements are in place to support cross-boundary working across geographical and commissioning areas, for example, with neighbouring health services or local authority teams | Modified | Agreements are in place to support cross-boundary working across geographical and commissioning areas, for example, with neighbouring health services and local authorities. |
| 120 | Joint visits between crisis team staff and professionals from other agencies take place when necessary | FG | H | Retained | Modified | Joint visits between service staff and professionals from other agencies take place when necessary |
| 121 | Assessments completed by the crisis team are understood and trusted by professionals in other teams or agencies to avoid unnecessary duplication of assessments | SI | H | Combined with standard no. 127 |  |  |
| 122 | The crisis team liaises with the patient’s GP, including them in decision making and correspondence | SI | H | Retained | Combined with no. 129 and modified | The service liaises with the person with dementia’s General Practitioner (GP). The service is explicit with GPs about what timely information is required in a referral, and what physical health checks should be undertaken prior to referral. The service includes GPs in decision making where relevant and through correspondence. |
| 123 | Complex cases involving several agencies discussed at higher meetings | SI | U | Prioritised for discussion at consensus workshop | Discarded |  |
| 124 | The team has good links with charities and the voluntary sector in the local area | SI, FG | H | Combined with standard no. 117 and 128 |  |  |
| 125 | Other services are aware of the existence of the team | SUI, CI, FG | N | Discarded |  |  |
| 126 | Team members explain clearly to service users and carers how they have become involved and why the service user or carer were referred to the team | SUI, CI | H | Retained | Combined with no. 15 and 23 |  |
| 127 | The team has good communication with other services involved in the care of the service user and carer | SUI, CI, FG | H | Combined with standard no. 121: The team has good communication with other services involved in the care of the service user and carer to avoid unnecessary duplication of assessments | Modified | The service has good communication with other services involved in the care of the person with dementia and their carers/families to avoid unnecessary duplication of assessments. |
| 128 | There are established pathways and policies for working with other agencies | FG | H | Combined with standard no. 117 and 124: There are established pathways and policies for working with all other relevant agencies, to include social services, charities and the voluntary sector. Other agencies and services have an accurate perception of the crisis team and its remit | Combined with no. 109 |  |
| 129 | Crisis teams are explicit with GPs about what information is required in a referral, and what physical health checks must be completed prior to referrals | FG | H | Retained | Combined with no. 122 |  |
| 130 | Crisis teams have good working relationships with GPs | FG | N | Discarded |  |  |
|  | **Resources – Team Base Environment** | | | | | |
| 131 | The crisis team have ownership or full use of their base and are able to make necessary changes to their environment | FG | N | Discarded |  |  |
| 132 | There is provision of IT resources and associated IT support appropriate to the needs of the crisis team | SI, FG | H | Combined with standard no. 137 and 138: There is provision of IT resources and associated IT support appropriate to the needs of the crisis team with access to computer systems and electronic notes to encourage working remotely, from various locations | Modified | There is provision of Information Technology (IT) resources and associated IT support appropriate to the needs of the service. This includes access to computer systems, including electronic notes, to enable working remotely from various locations. |
| 133 | The crisis team are provided with the space to complete paperwork and conduct telephone calls | SI, FG | H | Combined with standard no. 134 |  |  |
| 134 | The crisis team have access to a space large enough to facilitate MDT meetings | SI, FG | H | Modified and combined with standard no. 133: The crisis team have access to an appropriate space to facilitate MDT meetings, complete paperwork and conduct telephone calls | Modified | The service has access to appropriate space to facilitate Multi-disciplinary Team (MDT) meetings, and for staff to complete paperwork and conduct telephone calls of a confidential and/or sensitive nature. |
| 135 | The team can access medication to service users | SI | H | Modified: The team can access and dispense medication to service users | Combined with no. 159 and modified | Service staff review medication and monitor its effectiveness. Service staff have access to prescription of medication and are able to dispense it. |
| 136 | Team members have their own desks | SI | N | Discarded |  |  |
| 137 | Team members can access computer systems and electronic notes when working remotely | SI | H | Combined with standard no. 132 and 138 |  |  |
| 138 | Team members are able to work from various localities e.g. other bases within the Trust to reduce travel time to service users | SI | H | Combined with standard no. 132 and 137 |  |  |
|  | **Resources - Referrals** | | | | | |
| 139 | Service user flow through the service is monitored and evaluated | SI | H | Combined with standard no. 140: Service user flow should be measured for the purposes of service planning and all team members are made aware of this information | Combined with no. 42 and 87 |  |
| 140 | Team members are aware of the referral rates to the service | SI | H | Combined with standard no. 139 |  |  |
|  | **Assessment - Assessments** | | | | | |
| 141 | The team should use standardised assessments to assess service users when they are referred to the service | SI, S | H | Modified: The team should use standardised measures to assess and inform care planning. | Combined with no. 65 and 145 |  |
| 142 | The purpose and outcomes of assessments conducted by the team should be clearly explained to service users and carers | SUI, CI | H | Retained | Modified | The purpose and outcomes of assessments used by service staff are clearly explained to the person with dementia and their carers/families. |
| 143 | The team should provide specialist assessments such as cognitive assessments, mood, quality of life, carer burden, risk, and crisis | SI, SUI, CI, S | H | Combined with standard no. 145 |  |  |
| 144 | Assessments and visits are carried out at the service user’s home rather than in a clinic setting | SI, FG | M | Prioritised for discussion at consensus workshop | Combined with no. 66 |  |
| 145 | Assessment use should be tailored to the specific needs of the service user and carer, e.g. to consider anxiety, depression or mobility. Assessments should not be used if they are felt to be unsuitable for the individual needs of the service user, e.g. if they are unable or if it would provoke an emotional response unnecessarily | SI, SUI, CI | H | Combined with standard no. 143: Initial assessment includes a comprehensive risk assessment and must be tailored to the specific needs of the service user and carer, e.g. to also consider mood, cognitive ability, quality of life or carer burden. Assessments should not be used if they are felt to be unsuitable for the individual needs of the service user, e.g. if they are unable or if it would provoke an emotional response unnecessarily | Combined with no. 65 and 141 |  |
| 146 | Team members should find all relevant information about service user and carer e.g. preferences, life history, idiosyncrasies during the assessment process | SI, SUI, CI, FG | M | Prioritised for discussion at consensus workshop | Discarded |  |
|  | **Psychological Interventions** | | | | | |
| 147 | The crisis team provides proactive outreach to residential care homes and care assistants in the community with education, support and advice to prevent crises | SI, SUI, CI, FG | H | Combined with standard no. 148 and 151 |  |  |
| 148 | The crisis team can support other professionals with assessments and advice and prevention support | FG | H | Combined with standard no. 147 and 151 |  |  |
| 149 | The team provides education and support to carers to help them support the service user at home | SI, SUI, CI, FG | H | Combined with standard no. 150 and 152: The team provides education and support to carers to help them support the service user at home, which may include information about dementia, including basic information about what diagnosis the service user has and what the symptoms may include and signposting to available resources and services for service users and carers where relevant | Modified | Service staff provide information and education relevant to the specific dementia diagnosis, tailored to individual needs, to help carers/families support the person with dementia at home. |
| 150 | The team provide signposting to available resources and services for service users and carers | SI, SUI, CI, FG | H | Combined with standard no. 149 and 152 |  |  |
| 151 | The team engages in interventions to prevent crisis | SI | H | Combined with standard no. 147 and 148: The team engages in interventions to prevent crisis. That may include proactive outreach to residential care homes and care assistants in the community with education, support and advice and The crisis team can support other professionals with assessments and advice and prevention support | Combined with no. 156 and modified | Service staff engage in interventions to prevent further crisis; these may include assessment, advice and support for other professionals. |
| 152 | The team should provide information about dementia, including basic information about what diagnosis the service user has and what the symptoms may include | SUI, CI, FG | H | Combined with standard no. 149 and 150 |  |  |
| 153 | The team should provide interventions to reduce burden for service users and carers through providing practical problem-solving techniques | SUI, CI | H | Retained | Modified | Service staff provide interventions to improve quality of life for the person with dementia and their carers/families by providing practical assistance and problem solving techniques. |
| 154 | The team can access respite care facilities | SI, FG | H | Retained | Combined with no. 55 and modified | Service staff signpost and facilitate referrals to other services including respite care. |
| 155 | The team facilitates referrals to longer term services for the service user and carer | SI | H | Retained | Combined with no. 54 |  |
| 156 | The team provides secondary prevention of further crises | SI | H | Combined with standard no. 158: The team provides secondary prevention of further crises. This may include educating informal and formal carers on how to prevent reoccurrence of crisis | Combined with no 151 |  |
| 157 | The team provides an holistic approach, considering physical, mental, and social needs | FG | H | Retained | Modified | Service staff take an holistic approach, considering physical health, mental health, and social needs. |
| 158 | The team provides secondary prevention of crisis by educating informal and formal carers on how to prevent reoccurrence of crisis | FG | H | Combined with standard no. 156 |  |  |
|  | **Pharmacological Interventions** | | | | | |
| 159 | The team should review medication that the service user is prescribed | SI, FG | H | Modified: The team should review or be able to arrange for a review of medication that the service user is prescribed | Combined with no. 135 |  |
|  | **Onward Referral** | | | | | |
| 160 | Service users and carers are kept informed of negotiations between other teams and agencies providing onward care | SUI, CI, FG | H | Combined with standard no. 164. |  |  |
| 161 | Length of stay at the crisis team is defined and adhered to except in exceptional circumstances where flexibility is required. | SI | N | Discarded |  |  |
| 162 | Service users and carers are adequately prepared for discharge from the service and are involved in the decision to discharge | SUI, CI, FG | H | Modified: Service users and carers are adequately prepared for discharge from the service, are aware of how to re-access the team if necessary and are involved in the decision to discharge. Written and face-to-face information is offered. | Combined with no. 164 and modified | People with dementia and their carers/families are involved in the decision to discharge, are adequately prepared for discharge, and are aware how to re-access the service if necessary. Verbal and written information is offered which includes information about onward services organised by the crisis service. |
| 163 | Team members explain to the service users and carers which teams they are being referred to and why | SUI, CI | H | Combined with standard no. 164 |  |  |
| 164 | The team brokers onward support through negotiating referrals to longer term services to support the service user and carer | FG | H | Combined with standard no. 163: The team brokers onward support through negotiating referrals to longer term services to support the service user and carer. Service users and carers are kept informed of negotiations between other teams and agencies providing onward care. Team members explain to service users and carers which teams they are being referred to and why | Combined with no. 162 |  |
| 165 | Discharge from the service should be a clean break where service users and carers are aware that they will not receive further input from the team | FG | N | Discarded |  |  |
